# *PNL*: a software to build polygenic risk scores using a super learner approach based on PairNet, a Convolutional Neural Network

**DOI:** 10.1093/bioinformatics/btaf071

**Published:** 2025-02-14

**Authors:** Ting-Huei Chen, Chia-Jung Lee, Syue-Pu Chen, Shang-Jung Wu, Cathy S J Fann

**Affiliations:** Department of Mathematics and Statistics, Université Laval, Quebec, QC, G1V 0A6, Canada; Department of Pathology and Immunology, Washington University School of Medicine, St. Louis, MO, 63110, United States; Institute of Biomedical Sciences, Academia Sinica, Taipei, 115, Taiwan; Institute of Biomedical Sciences, Academia Sinica, Taipei, 115, Taiwan; Institute of Biomedical Sciences, Academia Sinica, Taipei, 115, Taiwan

## Abstract

**Summary:**

Polygenic risk scores (PRSs) hold promise for early disease diagnosis and personalized treatment, but their overall discriminative power remains limited for many diseases in the general population. As a result, numerous novel PRS modeling techniques have been developed to improve predictive performance, but determining the most effective method for a specific application remains uncertain until tested. Hence, we introduce a novel, versatile tool for building an optimized PRS model by integrating candidate models from multiple existing PRS building methods that use target population data and/or incorporating information from other populations through a trans-ethnic approach. Our tool, *PNL* is based on *PairNet* algorithm, a Convolutional Neural Network with low computation complexity through simple paring operation. In the case studies for asthma, type 2 diabetes, and vertigo, the optimal PRS model generated with *PNL* using only Taiwan biobank (TWB) data achieved Area Under the Curves (AUCs) that matched or improved the best results using other methods individually. Incorporating the UK Biobank data (UKBB) data further improved performance of *PNL* for asthma and type 2 diabetes. For vertigo, unlike the other diseases, individual method analysis showed that UKBB data alone generally produced lower AUCs compared to TWB data alone. As a result, incorporating UKBB data did not improve AUC with *PNL*, suggesting that increasing the number of candidate models does not necessarily result in higher AUC values, alleviating concerns about overfitting.

**Availability and implementation:**

The python code for *PairNet* algorithm incorporated in *PNL* is freely available on: https://github.com/FannLab/pairnet. An archived, citable version is stored on: https://doi.org/10.5281/zenodo.14838227.

## 1 Introduction

The inheritance of polygenic traits and complex diseases is governed by the interplay of multiple genetic variants. Polygenic risk scores (PRSs), which encapsulate an individual’s genetic predisposition to a particular trait or disease, have emerged as promising tools for early disease diagnosis, screening, and other clinical applications. Despite their potential, their predictive accuracy remains generally insufficient for a wide range of diseases in the broader population. In response, numerous PRS modeling methods have been developed in recent years to enhance their predictive capabilities.

Ma and Zhou (2021) provide a comprehensive review of existing PRS modeling techniques, and concurrently, new methods are continuously evolving. In essence, PRS modeling strategies encompass modeling the underlying genetic architecture, leveraging pleiotropic single nucleotide polymorphism (SNP) effects shared among the target disease and other traits or diseases, integrating SNP annotation and pathway information.

Since many methods rely on Genome-Wide Association Study (GWAS) summary statistics as input data, their effectiveness is closely linked to the statistical power of the GWAS, which is influenced by factors like sample size. Trans-ethnic PRS modeling techniques aim to enhance predictive performance in underrepresented populations, particularly in cases where GWAS summary statistics or sample sizes are limited.

Most PRS modeling techniques incorporate tuning parameters for model specifications. Consequently, model selection is necessary to identify the optimal PRS model from a set of multiple candidate models corresponding to different tuning values, using a specific criterion. Conventionally, when utilizing a particular PRS modeling technique and to ensure an unbiased result, a segment of *internal data* is allocated for model selection and another independent segment would be used to report the predictive performance of the optimal PRS model. The model selection process can become much more complicated when multiple PRS modeling techniques with different sets of tuning parameters are utilized.

From the user’s perspective, the objective is to attain a PRS model with the utmost predictive efficacy. Yet, determining *a priori* which PRS modeling method best suits a user’s specific application for a given trait or disease within a particular population poses a challenge. Moreover, different PRS modeling methods may offer distinct advantages and complement each other. Therefore, a tool that simplifies the model selection process and efficiently combines PRS models generated by multiple methods into an optimal PRS model is highly valuable. Our tool *PNL* was designed to fulfill this purpose.

*PNL* employs the *PairNet* algorithm (Jhang *et al.* 2019) as an ensemble learning approach to construct an optimal PRS model from multiple PRS candidates. *PairNet* was chosen for this task due to its unique ability to hierarchically aggregate information by progressively combining pairs of inputs across multiple convolutional layers, enabling robust binary response prediction, which is one of the main objectives of PRS applications. This hierarchical approach not only improves the capture of local dependencies but also enables the effective ensembling of long-range interactions. The PRS candidate models align with the input characteristics of *PairNet*, as they exhibit local dependencies for models generated using a set of tuning values ordered from least to most stringent, corresponding to the tuning parameters of a particular PRS building method. In contrast, the PRS candidates show weaker correlations for models generated by different PRS methods.

## 2 Materials and methods 

**Figure 1.**
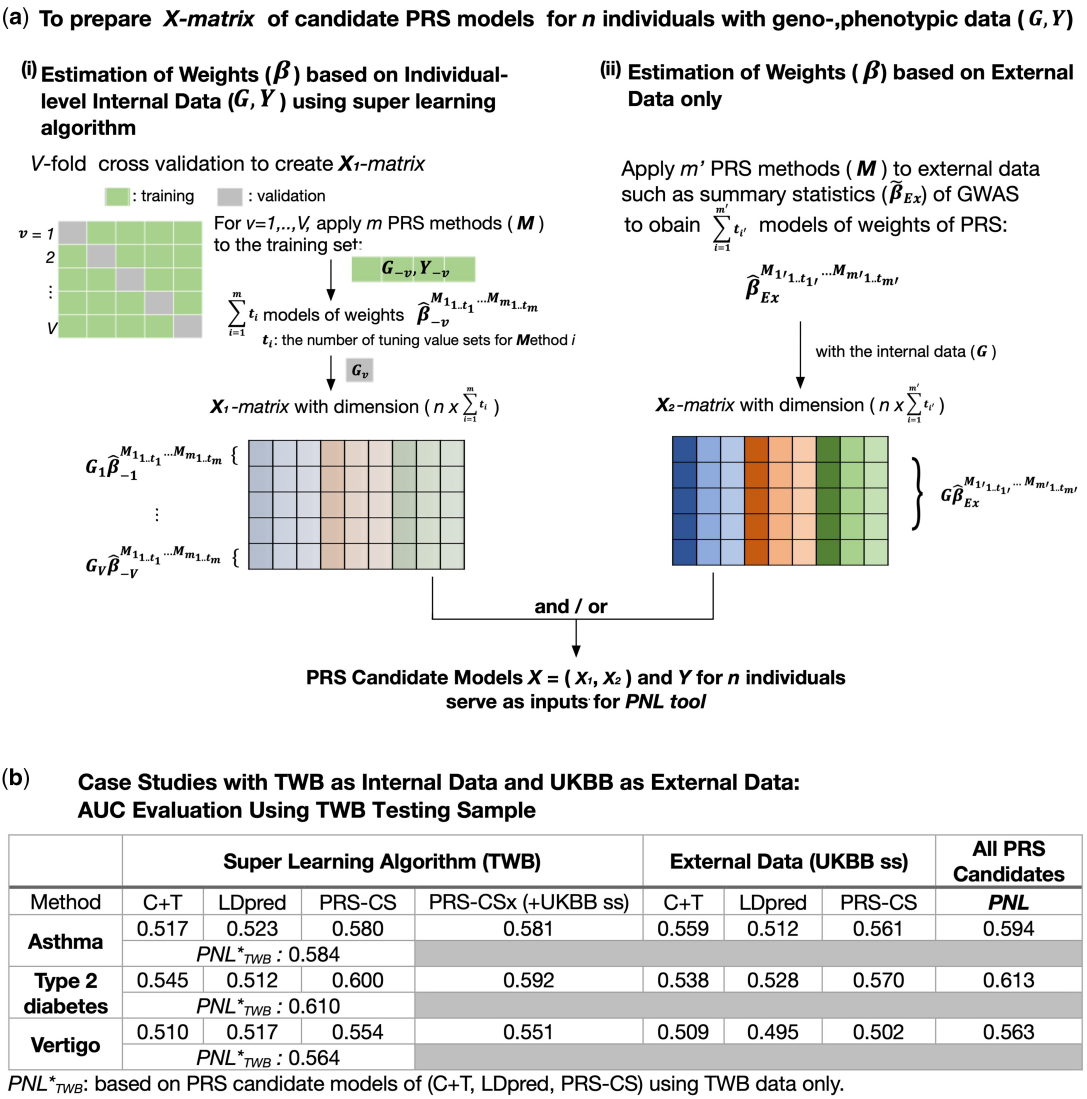
(a) A flowchart of input data preparation for *PNL* using (i) *internal data* including individual-level genotypic and phenotypic data for the target population and/or (ii) using *external data* such as external GWAS summary statistics, to generate PRS candidate models. (b) Analysis results of case studies on asthma, type-2 diabetes, and vertigo using TWB individual-level genotypic and phenotypic data as *internal data* and GWAS summary statistics derived from the UK Biobank data *external data*.

The input data for *PNL* comprises individual-level phenotypic values and PRS estimates from candidate models, which can be created by various PRS modeling techniques. Figure 1a summarizes the *PNL* framework.

Conventionally, most PRS modeling techniques use GWAS summary statistics to estimate the weights to compute PRS. Depending on each application study, one may choose to generate GWAS summary statistics internally by *internal data* including individual-level phenotypic and genotypic values, to use external GWAS summary statistics, denoted by *external data*, which is independent of *internal data*, or to apply both approaches. Correspondingly, the *PNL* framework illustrates the procedure for using *internal data* and *external data* to estimate the weights, as shown in Fig. 1a, i and ii, respectively.

For the first approach based on *internal data*, the super learning algorithm (Van der Laan *et al.* 2007) employs the V-fold cross-validation to prepare PRS candidate models, denoted by $X_{1}$ matrix. The n individuals of *internal data* (G,Y) are randomly partitioned into V equally sized or approximately equal sized subsets denoted by $s_{v}$, where v=1,..,V. Each subset $s_{v}$ serves as a validation sample and the remaining sets are considered as a training sample for estimating the weights for calculating PRS using the user’s specified PRS construction method, denoted by ${\hat{\beta }}_{\left(-v\right)}$. Then the PRS estimates for $s_{v}$ individuals are computed as $G_{v}{\hat{\beta }}_{\left(-v\right)}$. The identical estimation procedure is carried out for each subset respectively to generate PRS for all n individuals. Assume a user applies m different PRS building methods ($M_{i}$ denotes method i), each generating $t_{i}$ models (where i=1,…,m) corresponding to the number of tuning value sets for their respective tuning parameters. The resulting weights are denoted as ${\hat{\beta }}_{\left(-v\right)}^{M_{1_{1,.,t_{1}}},.,M_{m_{1,.,t_{m}}}}$, and all PRS values $G_{v}{\hat{\beta }}_{\left(-v\right)}^{M_{1_{1,.,t_{1}}},.,M_{m_{1,.,t_{m}}}}$ are combined row-wise for v=1,…,V to form $X_{1}$ with dimensions $n\times \sum_{i=1}^{m}t_{i}$.

In contrast, when the second approach, using only *external data*, is applied to estimate the weights (denoted by ${\hat{\beta }}_{Ex}$) from $m^{\prime }$ different PRS building methods, the PRS for the n individuals is computed as $G{\hat{\beta }}_{Ex}^{M_{1_{1,.,t_{1^{\prime }}}^{\prime }},.,M_{m_{1,.,t_{m^{\prime }}}^{\prime }}}$. The notations remain consistent with the first approach, with the distinction that the PRS building methods relying solely on external data may differ from those in the first approach, and thus the number of methods is denoted by $m^{\prime }$. The resulting PRS matrix $X_{2}$ is formed with dimensions $n\times \sum_{i^{\prime }=1}^{m^{\prime }}t_{i^{\prime }}$.

Assuming a total of C PRS candidate models are generated for n individuals, where, for instance, using both approaches results in $C=\sum_{i=1}^{m}t_{i}+\sum_{i^{\prime }=1}^{m^{\prime }}t_{i^{\prime }}$. The combined PRS matrix, $X=(X_{1},X_{2})$, along with the corresponding binary responses, forms the data matrix Z=(Y,X), with dimensions n×(C+1). The n samples are randomly divided into training, validation, and testing datasets. The training and validation sets are used to train the model and tune hyperparameters in the *PairNet* algorithm to aggregate the C PRS candidate models and produce an optimal predictor for the studied trait or disease. The testing dataset is then used to compute the AUC (Area Under the Curve), providing a measure of the predictive performance of the optimal PRS model.

## 3 Case studies

To demonstrate how to use *PNL*, we constructed PRS models for asthma, type-2 diabetes, and vertigo respectively using Taiwan biobank (TWB) data based on a similar data preparation procedure in Lee *et al.* (2022). The TWB dataset was considered as *internal data* including individual-level phenotypic and genotypic information, along with the GWAS summary statistics derived from the UK Biobank data (UKBB) Neale Lab (http://www.nealelab.is/blog/2017/7/19/rapid-gwas-of-thousands-of-phenotypes-for-337000-samples-in-the-uk-biobank), considered as external data. Detailed descriptions for the analysis procedure can be found in the Supplementary Materials.

In brief, to prepare PRS candidate models, the super learning algorithm in Fig. 1a, i using the *internal data* approach was applied to four PRS modeling techniques including C+T implemented by Purcell *et al.* (2007), LDpred (Vilhjálmsson *et al.* 2015), PRS-CS (Ge *et al.* 2019), and PRS-CSx (Ruan *et al.* 2022), where PRS-CSx integrating signals from UKBB GWAS summary statistics. In addition, the aforementioned PRS modeling techniques except for PRS-CSx were applied to UKBB GWAS summary statistics to prepare PRS candidate models using the approach based on *external data*  Fig. 1a, ii. Thus, the number of PRS building techniques used in the first approach is m=4, and in the second approach, it is $m^{\prime }=3$. For a brief description of the methods, C+T selects independent SNPs by pruning based on linkage disequilibrium (LD) and applying a significance threshold. LDpred refines this with a Bayesian framework, accounting for LD in SNP effect sizes. PRS-CS further improves accuracy using a continuous shrinkage prior, and PRS-CSx extends PRS-CS by incorporating multi-population data to enhance prediction across ancestries.

Figure 1b illustrates the predictive performance (AUC) for the Taiwanese population using the TWB dataset, applying each of the aforementioned PRS modeling techniques to asthma, type-2 diabetes, and vertigo, respectively. As described above, a testing dataset from the TWB sample was randomly selected to report AUC performance, while the remaining samples were used for PRS model training and tuning. For the three case studies, using only the TWB data to generate PRS candidate models, the optimal PRS model with *PNL* achieved AUCs of 0.584, 0.610, and 0.564 for asthma, type 2 diabetes, and vertigo, respectively. These AUCs are comparable to or exceed the best performances from the marginal analysis of the three methods (C+T, LDpred, PRS-CS) using TWB data alone. When incorporating PRS candidate models from UKBB data, *PNL* further improves predictive performance for asthma and type 2 diabetes. For vertigo, unlike the other diseases, individual method analysis showed that UKBB data alone generally produced lower AUCs compared to TWB data alone. As a result, incorporating UKBB data did not improve AUC with *PNL*. This indicates that increasing the number of candidate models does not necessarily result in higher AUC values, alleviating concerns about overfitting.

## 4 Conclusion

*PNL* is a Python package designed to build an optimal PRS model by integrating outputs from multiple PRS modeling techniques. This enables users to leverage the strengths of various approaches, including trans-ethnic methods, to enhance predictive performance for the trait or disease being studied. Since the *PairNet* algorithm can only handle binary responses, the applications of *PNL* are also limited to binary response scenarios.

## Supplementary Material

btaf071_Supplementary_Data

## Data Availability

The data including individual genotypes and phenotypes of TWB participants underlying this article were provided by Taiwan Biobank under licence / by permission.  A detailed description of TWB data availability and the application process can be found at https://www.biobank.org.tw/english.php.

## References

[btaf071-B1] Ge T , Chen C-Y, Ni Y et al Polygenic prediction via Bayesian regression and continuous shrinkage priors. Nat Commun 2019;10:1776. 30992449 10.1038/s41467-019-09718-5 PMC6467998

[btaf071-B2] Jhang Y-J , Chu Y-C, Tai T-M et al Sensor based dynamic hand gesture recognition by pairnet. In: *2019 International Conference on Internet of Things (iThings) and IEEE Green Computing and Communications (GreenCom) and IEEE Cyber, Physical and Social Computing (CPSCom) and IEEE Smart Data (SmartData), Atlanta, GA, USA*. New York, NY, USA: IEEE, 2019, 994–1001.

[btaf071-B3] Lee C-J , Chen T-H, Lim AMW et al Phenome-wide analysis of Taiwan biobank reveals novel glycemia-related loci and genetic risks for diabetes. Commun Biol 2022;5:1175. 36329257 10.1038/s42003-022-04168-0 PMC9633758

[btaf071-B4] Ma Y , Zhou X. Genetic prediction of complex traits with polygenic scores: a statistical review. Trends Genet 2021;37:995–1011. 34243982 10.1016/j.tig.2021.06.004 PMC8511058

[btaf071-B5] Purcell S , Neale B, Todd-Brown K et al Plink: a tool set for whole-genome association and population-based linkage analyses. Am J Hum Genet 2007;81:559–75. 17701901 10.1086/519795 PMC1950838

[btaf071-B6] Ruan Y , Lin Y-F, Feng Y-CA et al; Stanley Global Asia Initiatives. Improving polygenic prediction in ancestrally diverse populations. Nat Genet 2022;54:573–80. 35513724 10.1038/s41588-022-01054-7 PMC9117455

[btaf071-B7] Van der Laan MJ , Polley EC, Hubbard AE. Super learner. Stat Appl Genet Mol Biol 2007;6:Article25. 17910531 10.2202/1544-6115.1309

[btaf071-B8] Vilhjálmsson BJ , Yang J, Finucane HK et al; Schizophrenia Working Group of the Psychiatric Genomics Consortium, Discovery, Biology, and Risk of Inherited Variants in Breast Cancer (DRIVE) study. Modeling linkage disequilibrium increases accuracy of polygenic risk scores. Am J Hum Genet 2015;97:576–92. 26430803 10.1016/j.ajhg.2015.09.001 PMC4596916

