# Supplementary material for: PNL: a software to build polygenic risk scores using a super learner approach based on PairNet, a Convolutional Neural Network
Source: Bioinformatics. 2025 Feb 14;41(2):btaf071. doi: 10.1093/bioinformatics/btaf071 (PMC11879176; doi:10.1093/bioinformatics/btaf071)
Supplement: btaf071_Supplementary_Data [file btaf071_supplementary_data.pdf]

Supplementary information

1. Data preparation

1.1 Data source and format

internal data : TWB(plink format)
External data : UKB(summary statistics)

Data	UKB	TWB
Data Format	summary statistics	genotype
Case/contral (asthma)	39049/298110	3633 / 89616
Case/contral (type-2 diabetes)	2133/335026	5084 / 79705
Case/contral (vertigo)	2251/454,097	6199/ 54587
asthma SNP Count	10,894,596	5,929,384
type-2 diabetes SNP Count	10,894,596	5,928,690
vertigo SNP Count	11,831,932	16,211,759
Version	37	38

Note: Information for case-control number of type-2 diabetes and asthma in UKB data was found at http://www.nealelab.is/uk-biobank

1.2 Data processing

TWB

Using V-fold cross-validation to prepare polygenic risk score (PRS) candidate models for TWB individuals using TWB individual-level phenotypic and genotypic data.
Case-control sample sizes of each dataset in cross-validation for each of studied diseases.

- asthma:

	set1	set2	set3	set4	set5
case	727	727	727	726	726
control	17924	17923	17923	17923	17923

- type-2 diabetes:

	set1	set2	set3	set4	set5
case	1017	1017	1017	1017	1016
control	15941	15941	15941	15941	15941

- vertigo:

	set1	set2	set3	set4	set5
case	1240	1240	1240	1240	1239
control	10918	10918	10917	10917	10917

UKB

UKB summary statistics from https://pheweb.org/UKB-Neale/pheno/20002_1111 (asthma) & https://pheweb.org/UKB-Neale/pheno/20002_1223 (type-2 diabetes) & https://www.ebi.ac.uk/gwas/studies/GCST90043833 (vertigo)

UKB summary statistics were used for training weight functions in PRS models, and then the estimated weights were applied to TWB genotypes data to obtain PRS for TWB individuals.

2. Estimation of weights of PRS models

2.1 C+T method

Performed C+T analysis using plink 1.9 with the following parameters:

- **rsq** = 0.001,0.01,0.1
- **P**=0.05,0.01,0.005,0.001,0.0005,0.0001,0.00005,0.00001,0.000005,0.000001
- **kpcut** = 10000

```
plink \
        --bfile {input_prefix} \
        --allow-no-sex \
        --threads 10 \
        --clump {input.assoc} \
        --clump-p1 {params.p} \
        --clump-r2 {params.rsq} \
        --clump-kb {params.kpcut}\
        --out {output_prefix}
```

This process yields a '.clumped' file, which is then used with plink's 'score' function to obtain the '.profile' file. This '.profile' file contains the desired C+T PRS scores.

```
plink \
        --bfile {input_prefix} \
        --keep {input.sample} \
        --allow-no-sex \
        --extract {input.after_clump} \
        --score {input.score_data} \
        --out {output_prefix}
```

'after_clump' contains the retained SNPs from the clumped file, while 'score_data' is the organized clumped file, facilitating score computation. With the usage of 30 sets of parameter values for both UKB and TWB, a total of 60 C+T PRS scores were obtained.

2.2 LDpred

Used [LDpred v. 1.0.10] with the following parameters:

- **RHO** = '1.0000e-05','1.3000e-04', '1.7000e-03', '2.2000e-02', '2.8000e-01','-inf'

```
ldpred coord \
        --gf {params.ref_bed_prefix} \
        --ssf {params.ss_abspath} \
        --ssf-format CUSTOM \
        --rs SNP \
        --A1 A1 \
        --A2 A2 \
        --pos BP \
        --chr CHR \
        --pval P \
        --eff BETA \
        --N {params.train_n} \
        --eff_type LINREG \
        --vbim {input.vbim} \
        --out {output}
```

Because there are 6 sets of parameters for both UKB and TWB, a total of 12 LDpred PRS scores are generated.

2.3 PRS-CSx

Utilized [PRScsx-1.0.0] with the following parameters:

- **PARAM_PHI** = 1e+00,1e-01,1e-02,…,1e-09
- **SEED** = 42

```
python {params.PRScsx} \
        --ref_dir={params.ld} \
        --bim_prefix={params.input_prefix} \
        --sst_file={UKB_ss,TWB_ss} \
        --n_gwas={UKB_ss_n,TWB_ss_n} \
        --pop=EUR,EAS \
        --phi={wildcards.PARAM_PHI} \
        --a={params.a} \
        --b={params.b} \
        --chrom={params.chr} \
        --seed={params.seed} \
        --meta=True \
        --out_dir={params.out_dir} \
        --out_name={params.out_name}
```

With the two sets of summary statistics from TWB and UKB and the parameter $meta = True$, there would be three outputs at once. Considering 10 sets of parameter values, it generated 30 PRS-CSx scores

3. Software environment

- R

R	4.1.2
data.table	1.14.2

- python

python	3.6.15
snakemake	3.13.3
numpy	1.19.2
pandas	1.1.5
matplotlib	3.3.2
tqdm	4.62.3
scikit-learn	0.24.2
pytorch	1.7.0

4. Z matrix for super learning algorithm

Combining the 102 PRS candidate models generated above to create a Z matrix. All PRS models underwent normalization, and then the Z matrix was split into the training, validation,

and test sets with the following corresponding sample sizes for the two diseases.

- asthma:

	train	validation	test	total
case	2914	350	369	3633
control	71686	8974	8956	89616

- type-2 diabetes:

	train	validation	test	total
case	4091	477	516	5084
control	63741	8001	7963	79705

- vertigo:

	train	validation	test	total
case	5004	591	604	6199
control	43625	5487	5475	54587

5. PairNet

On the machine with Tesla P40, it takes 60 minutes in total to run type-2 diabetes, asthma, and vertigo.

5.1 PairNet github

https://github.com/FannLab/PairNet

5.2 Steps

- Import libraries
- Import PNL python package submodules
- Define Parameters
- load data
- Convert data to torch dataset format
- model train
- Obtain Results

5.3 Import libraries

```
import pandas as pd
import numpy as np
```

```
import matplotlib.pyplot as plt
from sklearn.metrics import roc_curve
from sklearn.metrics import roc_auc_score

import torch
import torch.nn.functional as F
import torch.utils.data as utils

from tqdm.auto import tqdm
import os
```

5.4 Import PNL python package submodules

```
from PairNet import PairNetClassifier
from utils_temp import (
       load_dataset,
       permute,
       split,
       arr_to_dataloader,
       accuracy_from_score,
       foward_batches,
       fit,
       predict
)
```

5.5 Define parameters

```
SEED = 42
EPOCHS = 20
MODEL_DIR = model_dir
GPU_ID = '0,1'

np.random.seed(SEED)
torch.manual_seed(SEED)
torch.backends.cudnn.benchmark = False
```

5.6 Load data

```
train_g, train_p, val_g, val_p, test_g, test_p = load_dataset(genotype_file,
sample_file,train_sample_file=train_id_file )
n_feature = train_g.shape[1]
```

5.7 Convert data to torch dataset format

```
train_loader = arr_to_dataloader(train_g, train_p, batch_size=256, shuffle=True)
val_loader = arr_to_dataloader(val_g, val_p, batch_size=256, shuffle=False)
test_loader = arr_to_dataloader(test_g, test_p, batch_size=256, shuffle=False)
```

5.8 Model train

```
#Using the PairNet model
model = PairNetClassifier(n_feature).cuda()
optimizer = torch.optim.AdamW(model.parameters(), 1e-3, weight_decay=1e-4)
#Defining the loss function
loss_fn = F.binary_cross_entropy
#Commencing training
performance , best_model_dict= fit(model, loss_fn, optimizer,
                  epochs=EPOCHS,
                  train_loader=train_loader, val_loader=val_loader,
test_loader=test_loader,
                  model_dir=MODEL_DIR)
```

5.9 Obtain results

Select the model that performs the best on the validation set.

```
best_model = PairNetClassifier(n_feature).cuda()
best_model.load_state_dict(best_model_dict)
```

Plot the graph displaying the change in loss.

```
fig_proc, ax_proc = plt.subplots()
# ax_proc.axvline(x=best_epoch, color='red')
for label, df in performance.groupby('stage'):
    ax_proc.plot(df.epoch.astype('int'), df.auroc, label=label)

ax_proc.legend()
ax_proc.set_title('Training Process')
ax_proc.set_xlabel('epoch')
ax_proc.set_ylabel('AUROC')

fig_proc.tight_layout()
```

```
fig_proc.savefig(snakemake.output.train)
```

Calculate the Area Under the Curve (AUC) and generate a plot.

```
val_pred, val_true = predict(val_loader, best_model)
val_fpr, val_tpr, val_thresholds = roc_curve(val_true, val_pred)
val_auroc = roc_auc_score(val_true, val_pred)



test_pred, test_true = predict(test_loader, best_model)
test_fpr, test_tpr, test_thresholds = roc_curve(test_true, test_pred)
test_auroc = roc_auc_score(test_true, test_pred)

fig_auroc, ax_auroc = plt.subplots()
ax_auroc.plot([0, 1], [0, 1], linestyle='--')
ax_auroc.plot(val_fpr, val_tpr, marker='.', label = 'validation')
ax_auroc.plot(test_fpr, test_tpr, marker='.', label = 'test')

ax_auroc.legend()
ax_auroc.set_title(f'AUROC val:{val_auroc:.3f}  test:{test_auroc:.3f}')
ax_auroc.set_ylabel('True Positive Rate')
ax_auroc.set_xlabel('False Positive Rate')

fig_auroc.tight_layout()
fig_auroc.savefig(snakemake.output.roc)
```
